# Degradation of *Sargassum crassifolium* Fucoidan by Ascorbic Acid and Hydrogen Peroxide, and Compositional, Structural, and In Vitro Anti-Lung Cancer Analyses of the Degradation Products

**DOI:** 10.3390/md18060334

**Published:** 2020-06-26

**Authors:** Tien-Chiu Wu, Yong-Han Hong, Yung-Hsiang Tsai, Shu-Ling Hsieh, Ren-Han Huang, Chia-Hung Kuo, Chun-Yung Huang

**Affiliations:** 1 Division of General Internal Medicine, Department of Internal Medicine, Kaohsiung Medical University Hospital, Kaohsiung Medical University, No. 100, Tzyou 1st Rd., Sanmin District, Kaohsiung City 80708, Taiwan; 960552@ms.kmuh.org.tw; 2 Department of Nutrition, I-Shou University (Yanchao Campus), No. 8, Yida Rd., Jiaosu Village, Yanchao District, Kaohsiung City 82445, Taiwan; yonghan@isu.edu.tw; 3 Department of Seafood Science, National Kaohsiung University of Science and Technology, No. 142, Haijhuan Rd., Nanzih District, Kaohsiung City 81157, Taiwan; yht@nkust.edu.tw (Y.-H.T.); slhsieh@nkust.edu.tw (S.-L.H.); 4 Department of Nursing, Mackay Medical College, No. 46, Sec. 3, Zhongzheng Rd., Sanzhi District, New Taipei City 25245, Taiwan; lisa68850@gmail.com

**Keywords:** ascorbic acid, anti-lung cancer, apoptosis, brown algae, fucoidan, human lung carcinoma A-549 cells, hydrogen peroxide, *Sargassum crassifolium*

## Abstract

Fucoidans possess multiple biological functions including anti-cancer activity. Moreover, low-molecular-weight fucoidans are reported to possess more bioactivities than native fucoidans. In the present study, a native fucoidan (SC) was extracted from *Sargassum crassifolium* pretreated by single-screw extrusion, and three degraded fucoidans, namely, SCA (degradation of SC by ascorbic acid), SCH (degradation of SC by hydrogen peroxide), and SCAH (degradation of SC by ascorbic acid + hydrogen peroxide), were produced. The extrusion pretreatment can increase the extraction yield of fucoidan by approximately 4.2-fold as compared to the non-extruded sample. Among SC, SCA, SCH, and SCAH, the chemical compositions varied but structural features were similar. SC, SCA, SCH, and SCAH showed apoptotic effects on human lung carcinoma A-549 cells, as illustrated by loss of mitochondrial membrane potential (MMP), decreased B-cell leukemia-2 (Bcl-2) expression, increased cytochrome c release, increased active caspase-9 and -3, and increased late apoptosis of A-549 cells. In general, SCA was found to exhibit high cytotoxicity to A-549 cells and a strong ability to suppress Bcl-2 expression. SCA also showed high efficacy to induce cytochrome c release, activate caspase-9 and -3, and promote late apoptosis of A-549 cells. Therefore, our data suggest that SCA could have an adjuvant therapeutic potential in the treatment of lung cancer. Additionally, we explored that the Akt/mammalian target of rapamycin (mTOR) signaling pathway is involved in SC-, SCA-, SCH-, and SCAH-induced apoptosis of A-549 cells.

## 1. Introduction

Lung cancer is one of the most commonly diagnosed cancers and is also one of the most deadly forms of cancer worldwide [1]. It is reported that 80% of lung cancers belong to the non-small-cell lung cancer (NSCLC) subtype that can be further divided into two classes: (1) lung adenocarcinoma (LUAD; 50%) and (2) lung squamous cell carcinoma (LUSC; 30%) [2,3]. In Taiwan, lung cancer is currently the most prevalent and most frequent cause of cancer-related mortality [4]. As such, it is crucial to develop novel agents and identify novel targets for the therapeutic treatment of lung cancer in order to improve patient outcomes.

Previous investigations reported that many anti-cancer drugs have drawbacks, such as side effects and toxicity [5]. Thus, there is a need for safer anti-cancer agents, particularly ones that can be manufactured using readily available naturally derived ingredients that cause no or minimal side effects. In recent years, researchers increasingly turned their efforts toward natural bioactive compounds due to their possible therapeutic activity in cancer at non-toxic levels [6]. Fucoidan is a water-soluble fucose-containing sulfated polysaccharide that is most commonly isolated from brown algae [7], and its α-l-fucose-enriched backbone also contains other monosaccharides, including glucose, xylose, galactose, and mannose [8]. Fucoidan possesses remarkable biological functions, including antioxidant, antitumor, anti-inflammatory, immunoregulatory, and antithrombotic activities [9]. These biological activities vary according to differences in the degree of sulfation, sulfation pattern, glycosidic branches, and molecular weight (MW) of fucoidan [9]. Low-molecular-weight (LMW) fucoidan is a highly sulfated fragment derived from fucoidan, and it received considerable attention due to its strong bioactivities with respect to anti-inflammatory, anticoagulant, antiangiogenic, antithrombosis, antioxidant, and anti-obesity effects [10,11]. In addition, LMW fucoidan is reported to be capable of modulating cell adhesion factor [12] and growth factor [13].

The process of extrusion comprises a short-duration, high-temperature bioreaction that involves mixing, heating, shearing, pressurizing, and shaping. During extrusion, the raw materials undergo mechanical shearing at high temperature with a very low moisture content and, thus, the properties of the extruded products, such as texture, microstructure, color, and flavor, are extensively modified [14]. Extrusion cooking provides numerous advantages, such as easy operation, continuous production, low manpower, high production yield, minimal waste, and a diversity of products [15]. Extruders are traditionally used to produce a wide variety of commonly consumed snacks, including corn curls, breadsticks, flatbreads, extruded corn ball, extruded puffed rice cereals, croutons, and breakfast cereals. Extrusion technology is also widely employed in the production of non-snack food products and other applications, such as biomass processing, and in the chemical, polymer, and energy industries [16]. Previous investigations indicated that extrusion can be successfully employed for the pretreatment of rice straw, which involves accelerating the saccharification of rice straw by enzymatic hydrolysis [17]. Fish scale is a good source for extraction of gelatin (a denatured form of collagen). However, it is known that fish scale is composed of collagen and hydroxyapatite, which are tightly linked together and difficult to separate. Extrusion was also adopted to pretreat fish scale to facilitate the separation of collagen and hydroxyapatite [15,18]. Soybean dregs can be pretreated by extrusion to decrease the quantity of insoluble dietary fiber (IDF) and increase soluble dietary fiber (SDF) in soybean residues [19]. Similarly, extrusion can be applied in the pretreatment of orange pomace to redistribute the IDF to SDF and to obtain a greater amount of soluble dietary fiber [20]. Moreover, the saccharification effect of lignocellulose can be improved by subjecting lignocellulosic biomass to bioextrusion pretreatment [21]. Therefore, we were interested in determining whether extrusion could be used to pretreat brown seaweeds in order to disrupt the natural anti-degradation barriers of seaweeds and enhance the release of polysaccharide from seaweed by water extraction alone. In the present study, we extracted fucoidan from *Sargassum crassifolium* pretreated by single-screw extrusion. The extracted fucoidan (native fucoidan, namely, SC) was then treated with different combinations of ascorbic acid (AA), hydrogen peroxide (H_2_O_2_), and AA + H_2_O_2_ to obtain degraded fucoidans. The composition, structure, and in vitro anti-lung cancer activity of native and degraded fucoidans were evaluated. This paper presents, for the first time to the authors’ knowledge, the in vitro anti-lung cancer activity of native and degraded fucoidans prepared from *S. crassifolium* pretreated by single-screw extrusion. In addition, we attempted to elucidate the underlying mechanisms involved in the fucoidan-induced lung cancer cell death. The result of this study may help to inform future research into the possible applications of degraded fucoidans as natural chemopreventive agents for the adjuvant treatment of cancer, especially lung cancer.

## 2. Results and Discussion

### 2.1. Preparation of Native and Degraded Fucoidans from S. crassifolium Pretreated by Single-Screw Extrusion

*S. crassifolium* consists of 0.98% lipid, 2.36% protein, 34.0% ash, and 62.7% carbohydrate (dry basis), according to previous research [22]. The predominant component in *S. crassifolium* is carbohydrate (more than 50%), which indicates that *S. crassifolium* is a good source for extraction of fucoidan. A single-screw extrusion process was used for pretreatment of brown algae before isolation of fucoidan. The extrusion parameters employed were raw material moisture content 35%, feed rate 10.4 kg/h, barrel temperature 115 °C, screw speed 360 rpm, and rounded die head with a diameter of 5 mm, and these parameters were developed previously by our laboratory [15] with minor modification. After extrusion, the algal extrudate was extracted using hot water (85 °C) for 1 h with shaking. Following the removal of alginic acid, the fucoidan extracts were precipitated by 50% ethanol and recovered by centrifugation using the method developed previously by our laboratory [23], and then the native fucoidan (namely, SC) was obtained. The extraction yields of fucoidans from non-extruded and single-screw-extruded *S. crassifolium* were 2.69 ± 0.97 and 11.3 ± 1.3 g/100 g, dry basis, respectively. These data indicate that the extrusion process augments the extraction yield of fucoidan by 4.2-fold (11.3/2.69 = 4.2), as compared to non-extruded sample. The extrusion process would, therefore, certainly provide a higher production rate and lower production cost in the commercial manufacture of fucoidan. The results of a similar experiment conducted by the authors suggested that the extrusion process also increases the extraction yield of gelatin from fish scale by a maximal value of 3.3-fold with 50 °C water extraction [15]. SC was, thus, utilized for further degradation experiments using different degradation reagent combinations including AA, H_2_O_2_, and AA + H_2_O_2_. Then, three degraded fucoidans, namely, SCA (degraded by AA), SCH (degraded by H_2_O_2_), and SCAH (degraded by AA + H_2_O_2_), were obtained. The native and degraded fucoidans were subsequently subjected to physicochemical, compositional, structural, and in vitro anti-lung cancer analyses.

### 2.2. Compositional and Physicochemical Analyses of Native and Degraded Fucoidans

Zhang and his coworkers utilized AA, H_2_O_2_, or a combination of AA + H_2_O_2_ to degrade raw polysaccharide from *Enteromorpha linza,* and they successfully obtained its lower-molecular-weight fractions [24]. In the present study, we followed the methods of Zhang et al. [24] to degrade SC with minor modification. The SC was degraded by 10 mM AA, 10 mM H_2_O_2_, or a mixed solution of 10 mM H_2_O_2_ and 10 mM AA for 16 h, respectively, and three degradation derivatives, namely, SCA, SCH, and SCAH, were obtained. To examine whether the degradation reagents could successfully degrade fucoidan, the intrinsic viscosities and molecular weights of native and degraded fucoidans were analyzed. Previous investigations suggested that the degradation of fucoidan solution resulted in a decline of its intrinsic viscosity [10,24]. As shown in Table 1, the viscosities of SC, SCA, SCH, and SCAH were 113.9% ± 2.4%, 78.6% ± 4.2%, 102.5% ± 0.9%, and 36.9% ± 2.6%, respectively, indicating that all degrading reagents could successfully degrade SC. Moreover, among SCA, SCH, and SCAH, SCAH had the lowest viscosity value, which suggests that the combination of AA and H_2_O_2_ may degrade SC more efficiently. High-performance liquid chromatography (HPLC) gel filtration is a powerful research tool that is used to accurately characterize the molecular weight distribution of polysaccharides [23]. Here, we applied an HPLC gel filtration method to analyze the molecular weight distributions of SC, SCA, SCH, and SCAH, as shown in Table 1. The results showed that the peak molecular weight (molecular weight of the highest peak) for SC was 427.8 kDa (the peak was in the molecular weight range of 188.7–1064 kDa) (peak area = 100.0%), while that for SCA was 455.1 kDa (the peak was in the molecular weight range of 216.2–898.9 kDa) (peak area = 50.3%) and 3.06 kDa (the peak was in the molecular weight range of 1.65–9.95 kDa) (peak area = 49.7%), that for SCH was 427.8 kDa (the peak was in the molecular weight range of 194.3–998.1 kDa) (peak area = 61.0%) and 3.14 kDa (the peak was in the molecular weight range of 1.90–15.54 kDa) (peak area = 39.0%), and that for SCAH was 487.1 kDa (the peak was in the molecular weight range of 264.5–956.4 kDa) (peak area = 49.2%) and 3.11 kDa (the peak was in the molecular weight range of 1.66–12.55 kDa) (peak area = 50.8%). When examining the molecular weight intervals of peak 2 (Table 1) of the three degraded fucoidans, it was found that SCA (1.65–9.95 kDa) possessed the lowest molecular weight, followed by SCAH (1.66–12.55 kDa), and then SCH (1.90–15.54 kDa). Moreover, it was also found that SCAH had the highest amount of low-molecular-weight fraction (50.8%), followed by SCA (49.7%), and then SCH (39.0%). However, the difference between SCAH and SCA is not obvious. These data show that SCA, SCH, and SCAH were partially degraded, with a high-molecular-weight fraction (peak molecular weight approximately 427.8–487.1 kDa) and a low-molecular-weight fraction (peak molecular weight approximately 3.06–3.14 kDa) coexisting in SCA, SCH, and SCAH. In this case, it was also found that 10 mM AA, 10 mM H_2_O_2_, or a mixed solution of 10 mM H_2_O_2_ and 10 mM AA could only partially degrade SC. For SCA, SCH, and SCAH, the separation of high-molecular-weight and low-molecular-weight fractions is feasible. However, the separation process is complicated, time-consuming, and costly, which might be detrimental for future commercialized applications. In addition, previous studies suggested that high-molecular-weight fucoidans (molecular weight approximately 735–750 kDa) exhibited good pharmacokinetic and tissue distribution after in vivo oral and topical applications [25,26]. Therefore, these degraded fucoidans (SCA, SCH, and SCAH) were directly utilized for further experiments. Since the molecular weight compositions of SC, SCA, SCH, and SCAH are varied, the biological functions, such as the anti-cancer activity of these fucoidans, warrant further investigation. Previous studies suggested that polyphenols are usually coextracted with fucoidans [23]. We also found that the coextracted polyphenols in SCA, SAH, and SCAH were diminished as compared to that of SC (Table 1), indicating that the polyphenols were also partially degraded by these degradation reagents. Previous studies revealed that continuous addition of H_2_O_2_ during the degradation reaction facilitates the generation of lower-molecular-weight products [27]. Thus, further elucidation of degradation reagents and degradation methods which can thoroughly degrade SC is needed. Previous studies revealed that the bioactive properties of fucoidan may vary depending on the molecular weight, sulfate content, sugar type, and monosaccharide composition [28,29,30]. Here, we analyzed the chemical and monosaccharide compositions of SC, SCA, SCH, and SCAH. The data depicted in Table 1 suggest that the total sugar contents of SCA, SCH, and SCAH ranged from 30.83% ± 0.21% to 41.70% ± 0.91% *(w*/*w*, dry basis), which were lower than that of SC (45.58% ± 0.80%). This observation was consistent with data reported by Hou et al. [31], which suggested that the total sugar content of fucoidan decreased with the reduction in molecular weight of fucoidan after degradation by hydrogen peroxide. A possible reason for this phenomenon may be the destruction of the sugar unit under the degradation process [31]. l-Fucose was found to be the predominant sugar unit in fucoidan, and the content of fucose may have a role in biological functions [32,33]. In addition, previous studies suggested that monosaccharides, such as l-fucose, have anticancer potential. It is documented that deficient fucosylation may play an important role in the pathogenesis of cancer. The supplementation of l-fucose could restore fucosylation in both in vitro and in vivo conditions and could alleviate cancer symptoms [34]. The fucose contents of SC, SCA, SCH, and SCAH were 27.31% ± 1.59%, 35.22% ± 2.79%, 20.08% ± 1.68%, and 30.08% ± 3.11%, respectively. Previous studies suggested that the fucose content of fucoidan increased with the reduction in molecular weight of fucoidan after degradation by hydrogen peroxide and ascorbic acid [35]. However, another report revealed that the fucose content of fucoidan decreased with the reduction in molecular weight of fucoidan after degradation by hydrogen peroxide [31]. Therefore, the precise effects of degradation conditions on the fucose content of fucoidan remain to be elucidated. In general, our data suggest that SCA had the highest fucose content. Thus, their anticancer potential, especially that of SCA, warrants further study. The presence of sulfate in fucoidan may be related to its biological functions [36,37]. We, thus, measured the sulfate contents of SC, SCA, SCH, and SCAH, and the percentages were 18.64% ± 1.43%, 13.67% ± 2.19%, 19.23% ± 0.83%, and 20.39% ± 3.28%, respectively. Our data suggest that the sulfate contents seem to be unrelated to the degradation treatments. These data are in line with previous reports suggesting that there is no relationship between the sulfate content of fucoidan and the reduction in molecular weight of fucoidan after degradation by hydrogen peroxide + ascorbic acid or hydrogen peroxide [24,31]. The monosaccharide compositions of the fucoidans were analyzed, and the data are presented in Table 1. For SC, fucose, galactose, glucuronic acid, and galacturonic acid were the major neutral sugar constituents, and the minor sugar units were mannose and xylose. After degradation, the amounts of glucuronic acid and galacturonic acid in degraded fucoidans seemed to have decreased. In summary, intrinsic viscosity and molecular weight analyses showed that all of the tested degradation reagents could degrade SC. The native and degraded fucoidans contained different amounts of total sugar, fucose, and sulfate, and they had dissimilar monosaccharide compositions. Since differences in physicochemical characteristics among SC, SCA, SCH, and SCAH were found, the biological functions of these fucoidan extracts warrant further investigation.

### 2.3. Structural Analyses of Native and Degraded Fucoidans

**Figure 1:**
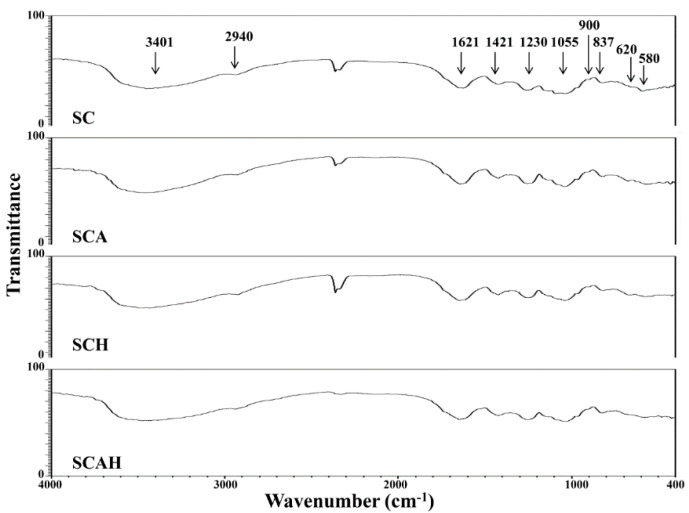
Fourier-transform infrared (FTIR) spectra for SC, SCA, SCH, and SCAH. The characteristic peaks are labeled.

Fourier-transform infrared (FTIR) and nuclear magnetic resonance (NMR) spectroscopy techniques were utilized to characterize the structures of native and degraded fucoidans. The FTIR spectra of SC, SCA, SCH, and SCAH within the range of 4000–400 cm^−1^ are depicted in Figure 1. IR bands at 3401, 2940, 1230, and 1055 cm^−1^ are due to the presence of OH and H_2_O stretching vibration, mainly by C–H stretching of the pyranoid ring or the C-6 group of fucose and galactose units, the S=O stretching of sulfates, and the C–O–C stretching vibrations in rings or C–O–H in the glucosidal bond [38,39]. Strong absorption bands were observed at 1621 and 1421 cm^−1^, which can be attributed to scissoring vibration of H_2_O and in-plane ring CCH, COH, and OCH vibrations, characterized by the absorption of polysaccharide [38,39,40]. The absorption bands at 900 and 837 cm^−1^ can be attributed to the presence of C1–H bending in the β-anomeric link (probably galactose) and equatorial C–O–S bending vibration of sulfate substituents at the axial C-4 position [41]. The bands near 620 and 580 cm^−1^ could be assigned to the symmetric and anti-symmetric O=S=O deformations [42]. Due to the resemblance of the FTIR spectra in SC, SCA, SCH, and SCAH, the structural aspects of these sulfated polysaccharides were not obviously altered by the degrading treatments. NMR spectroscopy is usually adopted to analyze polysaccharides with complex structures [43]. In this study, we utilized ^1^H-NMR and ^13^C-NMR spectra to examine the structural features of SC, SCA, SCH, and SCAH. The ^1^H-NMR spectra (Figure S1A, Supplementary Materials) for SC, SCA, SCH, and SCAH revealed that the signals between 5.5 and 5.0 ppm corresponded to l-fucopyranosyl units [44], the signal at 4.46 ppm, which was obvious in SCA and SCAH, indicates H-2 of a 2-sulfated fucopyranose residue [44], and the signal arising from 4.13 ppm at 4[H] represents 3-linked α-l-fucose [40]. The signals with ppm of 4.07/3.95 (6[H]/6′[H]) correspond to a (1-6)-β-d-linked galacton [45]. The signals between 3.9 and 3.6 ppm may be due to the presence of mannitol [46,47], which is often coextracted with fucoidan. The signal at 3.78 ppm (3[H]) (most notably in SC) can be attributed to 4-linked β-d-galactose, whereas the signal at 3.72 ppm can be assigned to (4[H]) 2,3-linked α-β-mannose [40]. The signals between 2.21 and 2.14 ppm may be tentatively assigned to methyl protons in *O*-acetyls [40,48], which are often present in algal polysaccharides [48]. The signal obtained at 1.92 ppm (1[H]) signifies alkyl at a sulfonyl-attached proton, and 1.23 ppm (6[H]) indicates an alkane proton in two methyl groups [49]. Due to the similar structural features observed among SC, SCA, SCH, and SCAH (Figure S1, Supplementary Materials) by ^1^H-NMR analysis, it appears that the structural characteristics of fucoidan were not evidently altered by the treatments with degrading reagents. The ^13^C-NMR spectra (Figure S1B, Supplementary Materials) for SC, SCA, SCH, and SCAH revealed that the prominent signal at 101.6 ppm and peaks between 65 and 80 ppm correspond to (1-6)-β-d-linked galacton [45].The signal at 100.3 ppm can be assigned to (1,3)-linked α-l-fucopyranose residues [47]. The signals at 62.0 ppm 66.7 ppm were attributed to β-d-galactopyranose residues [50]. The signals at 19–20 ppm revealed the presence of *O*-acetyl groups [51]. Due to the similar structural features observed among SC, SCA, SCH, and SCAH (Figure S1B, Supplementary Materials) by ^13^C-NMR analysis, it appears that the structural characteristics of fucoidan were not obviously altered by the treatments with degrading reagents. In summary, the FTIR, ^1^H-NMR, and ^13^C-NMR data provide evidence that SC, SCA, SCH, and SCAH possess characteristic structural features of fucoidan. As SCA, SCH, and SCAH are partially degraded derivatives of SC, these four compounds exhibited similar structural features. Therefore, in our case, the differences in bioactivities among SC, SCA, SCH, and SCAH may not be predominantly attributed to their structure since they share similar structural features.

### 2.4. SC, SCA, SCH, and SCAH Exhibited Cytotoxic Effects on A-549 Cells

**Figure 2:**
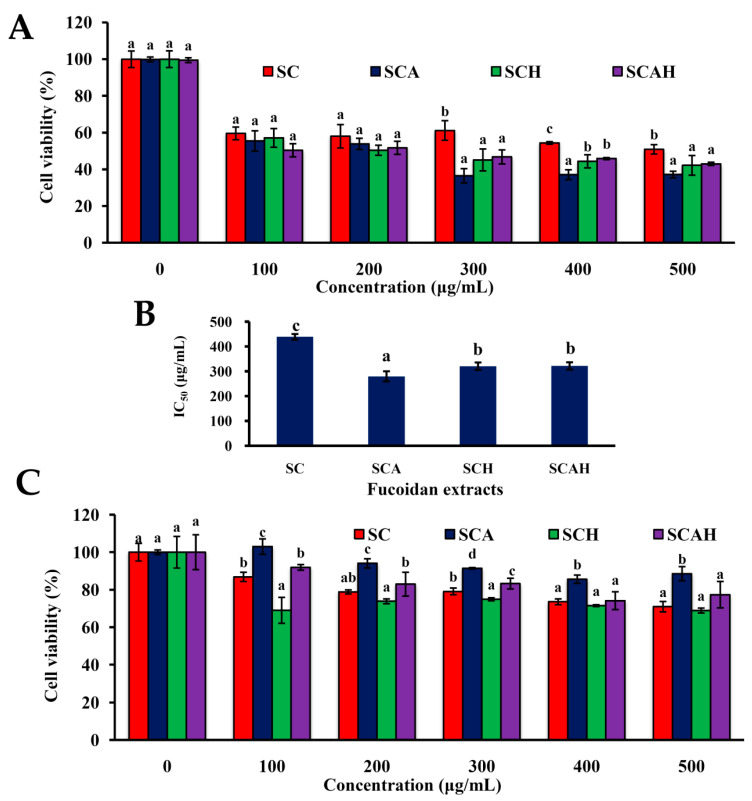
Effects of SC, SCA, SCH, and SCAH on cell viabilities of A-549 and BEAS-2B cells: (**A**) A-549 cells were co-incubated with various concentrations of SC, SCA, SCH, and SCAH for 48 h, and cell viability was assessed. Bars in the same treating concentration bearing different letters (in a, b, and c) significantly differ at the level of 0.05; (**B**) bar graphs show the half maximal inhibitory concentration (IC_50_) values (the inhibitory concentrations at 50% growth of A-549 cells) of SC, SCA, SCH, and SCAH as determined for (**A**). Bars bearing different letters (in a, b, and c) significantly differ at the level of 0.05; (**C**) BEAS-2B cells were co-incubated with various concentrations of SC, SCA, SCH, and SCAH for 48 h, and the cell survival was evaluated. Bars in the same treating concentration bearing different letters (in a, b, c, and d) significantly differ at the level of 0.05. Experiments were repeated three times.

The inhibition of cancer cell proliferation can be utilized to evaluate the potential anti-cancer ability of novel substances [33]. Here, we utilized an in vitro model (A-549 cells) to monitor the anti-lung cancer effects of the native and degraded fucoidans. In the preliminary experiments, A-549 cells were treated with 500 μg/mL of native and degraded fucoidans for 24, 48, or 72 h. It was found that a treatment time of 48 h was optimal for the induction of cytotoxicity in A-549 cells. For the purpose of ensuring consistency, the treatment time of cells was, thus, set to 48 h in all cellular experiments. As shown in Figure 2A, all fucoidans (SC, SCA, SCH, and SCAH) decreased the ratios of live A-549 cells, and SCA, SCH, and SCAH exhibited greater cytotoxic effects on A-549 cells than SC. Moreover, as shown in Figure 2B, among SCA, SCH, and SCAH, SCA showed cytotoxicity to A-549 cells with the lowest half maximal inhibitory concentration (IC_50_) value, indicating that SCA exhibited the greatest cytotoxicity to A-549 cells. These results indicate that degraded fucoidans may have stronger inhibitory effects on A-549 cells as compared to native fucoidan. BEAS-2B is a non-cancerous bronchial epithelial cell line, which can be used to represent normal human lung cells [52]. Additionally, we conducted a similar experiment utilizing BEAS-2B cells in an effort to determine whether or not these fucoidans exerted toxic effects on normal cells. The results suggest that SC and SCH exhibited the greatest cytotoxicities to BEAS-2B cells, followed by SCAH, and the least cytotoxic was SCA (Figure 2C). In addition, it was found that, at the concentration of 200 μg/mL, A-549 cells showed survival rates of approximately 50.4–58.0% and BEAS-2B cells showed survival rates of approximately 73.8–94.1% following exposure to each of the fucoidan extracts. Therefore, a concentration of 200 μg/mL was chosen for all of the tested fucoidans, and a treatment duration of 48 h was used for further in vitro anti-lung cancer experiments. Interestingly, SCA was highly cytotoxic to A-549 cells (survival rate 53.8% ± 3.1%), but only had a weak cytotoxic effect on BEAS-2B cells (survival rate 94.1% ± 2.5%) at a dose of 200 μg/mL and a treatment duration of 48 h (Figure 2A,C), indicating that SCA may be a better anti-lung tumor candidate. Taken together, all of the tested fucoidan extracts exhibited growth suppression of A-549 cells. SCA was shown to be a better choice as an anti-lung cancer agent due to its strong cytotoxic effect on A-549 cells and its low cytotoxic impact on normal lung cells.

### 2.5. SC, SCA, SCH, and SCAH Decreased Mitochondrial Membrane Potential (MMP) in A-549 Cells

**Figure 3:**
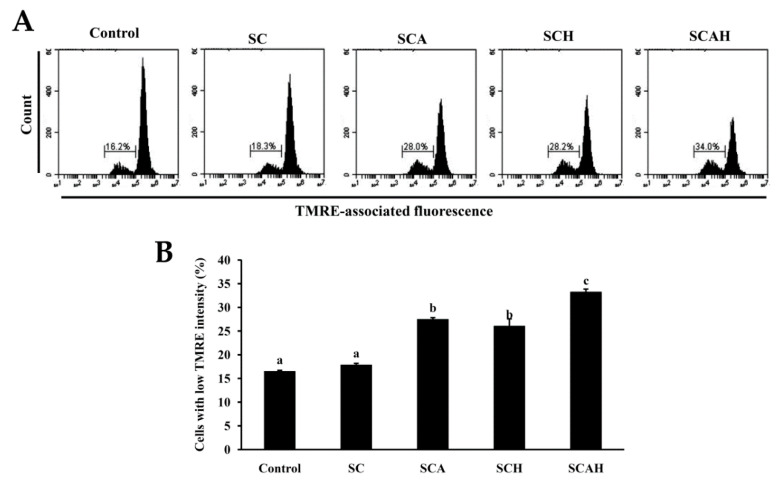
Effects of SC, SCA, SCH, and SCAH treatments on mitochondrial membrane potential (MMP) of A-549 cells. A-549 cells were treated without and with 200 μg/mL SC, SCA, SCH, and SCAH for 48 h, and MMP was determined by tetramethylrhodamine ethyl ester (TMRE) staining and flow cytometry: (**A**) histograms; (**B**) bar chart. Experiments were repeated three times. Bars bearing different letters (in a, b, and c) significantly differ at the level of 0.05.

Two fundamental pathways, namely, the intrinsic pathway (the mitochondria pathway) and the extrinsic pathway (death receptor pathway), are involved in cellular apoptosis [53]. The intrinsic pathway operates several processes involving the loss of mitochondrial membrane potential (MMP), release of cytochrome c, and formation of an apoptotic complex in which caspase-9 and caspase-3 are activated [54]. Mitochondrial dysfunction is suggested to play a central role in the mitochondria-dependent apoptotic pathway. Generally, loss of MMP can be regarded as an early event in the apoptotic process [55]. Thus, we evaluated the effects of SC, SCA, SCH, and SCAH on the MMP of A-549 cells. A potentiometric fluorescent tetramethylrhodamine ethyl ester (TMRE) dye, which is a cell-permeable, positively charged dye, can stably accumulate in active mitochondria due to their relatively negative charge. The loss of MMP may result in a decline of TMRE accumulation in mitochondria [22]. A statistically significant reduction in MMP was observed in A-549 cells following exposure to SCA, SCH, and SCAH at a dose of 200 μg/mL for 48 h, as compared to that of the untreated control (Figure 3). The greatest loss of MMP was induced by SCAH, followed by SCA and SCH, and SC had a similar effect on MMP as compared to the untreated control. These data indicate that degraded fucoidans (SCA, SCH, and SCAH) have a strong capability to induce loss of MMP in A-549 cells.

### 2.6. SC, SCA, SCH, and SCAH Decreased B-cell leukemia-2 (Bcl-2) Expression of A-549 Cells

**Figure 4:**
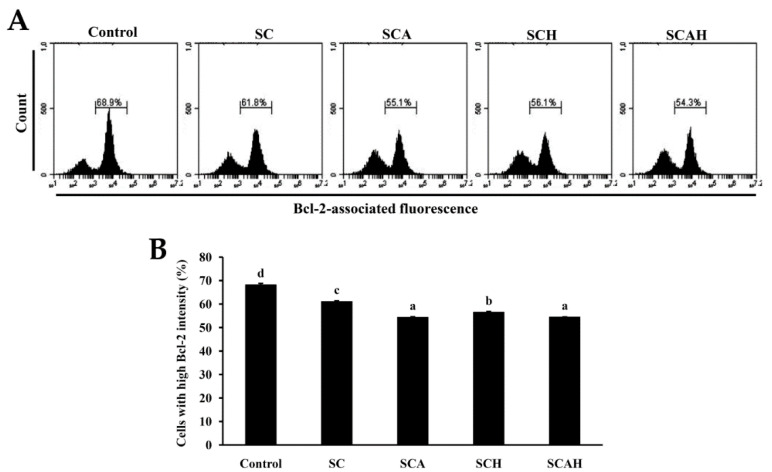
Effects of SC, SCA, SCH, and SCAH treatments on B-cell leukemia-2 (Bcl-2) expression in A-549 cells. A-549 cells were treated without and with 200 μg/mL SC, SCA, SCH, and SCAH for 48 h, and fluorescence histograms of immunolabeled Bcl-2 were determined by flow cytometry: (**A**) histograms; (**B**) bar chart. Experiments were repeated three times. Bars bearing different letters (in a, b, c, and d) significantly differ at the level of 0.05.

Bcl-2 belongs to the anti-apoptotic class of B-cell leukemia-2 (Bcl-2) gene product family proteins, and it is proposed to block MMP depolarization, which then retards the activation of various death effectors, such as release of cytochrome c, apoptosis-inducing factor (AIF), and Smac/Diablo [56]. In contrast, the suppression of Bcl-2 expression brings about cellular apoptosis. The effects of SC, SCA, SCH, and SCAH on Bcl-2 expression in A-549 cells were examined, and the data are shown in Figure 4. A statistically significant decrease in the level of Bcl-2 was observed in A-549 cells following exposure to SC, SCA, SCH, and SCAH at a dose of 200 μg/mL for 48 h as compared to the untreated control. Among the fucoidans tested, SCA and SCAH exhibited the greatest suppression of Bcl-2. In addition, the degraded fucoidans (SCA, SCH, and SCAH) appeared to have stronger activity in suppressing Bcl-2 expression in A-549 cells compared with SC.

### 2.7. SC, SCA, SCH, and SCAH Increase Cytochrome C Release of A-549 Cells

**Figure 5:**
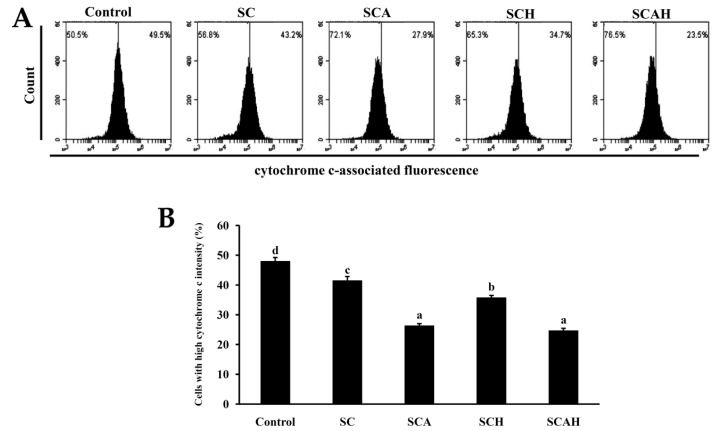
Effects of SC, SCA, SCH, and SCAH treatments on the amount of cytochrome c release in A-549 cells. A-549 cells were treated without and with 200 μg/mL SC, SCA, SCH, and SCAH for 48 h, and flow-cytometric profiles of immunolabeled cytochrome c were determined: (**A**) histograms; (**B**) bar chart. Experiments were repeated three times. Bars bearing different letters (in a, b, c, and d) significantly differ at the level of 0.05.

Previous investigations suggested that a decline in MMP results in matrix condensation and exposure of cytochrome c to the intermembrane space, which then facilitates cytochrome c release, resulting in apoptotic death [57]. Here, the effects of SC, SCA, SCH, and SCAH exposure on cytochrome c release in A-549 cells were examined, and the data are shown in Figure 5. A statistically significant decrease in the high fluorescence level of cytochrome c was observed in A-549 cells after exposure to SC, SCA, SCH, and SCAH at a dose of 200 μg/mL for 48 h as compared to the untreated control. Among the fucoidans tested, SCA and SCAH diminished cytochrome c to the greatest extent. In addition, the degraded fucoidans (SCA, SCH, and SCAH) appeared to exhibit stronger activity in the augmentation of cytochrome c release in A-549 cells compared with SC.

### 2.8. SC, SCA, SCH, and SCAH Increase Active Caspase-9 and -3 of A-549 Cells

**Figure 6:**
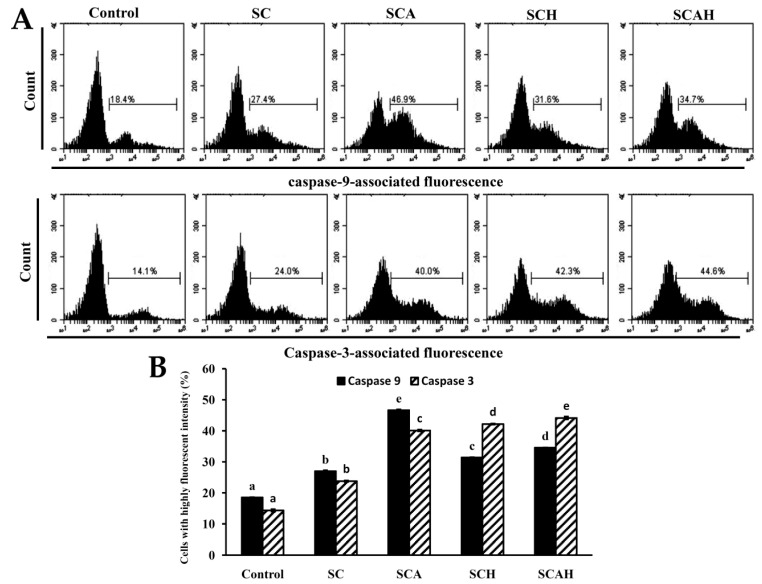
Effects of SC, SCA, SCH, and SCAH treatments on the activation of caspase-9 and -3 in A-549 cells. A-549 cells were treated without and with 200 μg/mL SC, SCA, SCH, and SCAH for 48 h, and the flow-cytometric profiles of immunolabeled caspase-9 and -3 were determined: (**A**) histograms; (**B**) bar chart. Bars in the same caspase-9 or caspase-3 group bearing different letters (in a, b, c, d, and e) significantly differ at the level of 0.05. Experiments were repeated three times.

In the mitochondrion-dependent pathway, the release of cytochrome c from the mitochondrial intermembrane space triggers the formation of an apoptosome, which induces the activation of caspase-9 and caspase-3 [58]. In the present study, the effects of SC, SCA, SCH, and SCAH on the activation of caspase-9 and -3 in A-549 cells were evaluated. The results shown in Figure 6 suggest that a statistically significant increase in the levels of active caspase-9 and -3 occurred in A-549 cells after exposure to SC, SCA, SCH, and SCAH at a dose of 200 μg/mL for 48 h as compared to the untreated control. Among the fucoidans tested, SCA exhibited the greatest effect on the upregulation of caspase-9, whereas SCAH induced the largest upregulation of caspase-3. In addition, the degraded fucoidans (SCA, SCH, and SCAH) showed stronger activity in terms of activation of caspase-9 and -3 in A-549 cells compared with SC.

### 2.9. SC, SCA, SCH, and SCAH Induce Apoptosis of A-549 Cells

**Figure 7:**
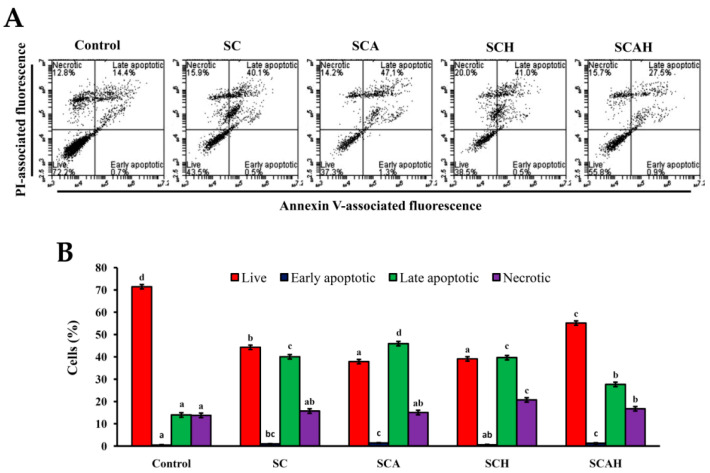
SC, SCA, SCH, and SCAH induce apoptosis in A-549 cells. Flow-cytometric analysis of Annexin V-fluorescein isothiocyanate (FITC)/propidium iodide (PI)-stained A-549 cells treated without and with 200 μg/mL SC, SCA, SCH, and SCAH for 48 h: (**A**) histograms; (**B**) bar chart. Bars in the same cell population bearing different letters (in a, b, c, and d) significantly differ at the level of 0.05. Experiments were repeated three times.

Once caspase-3 is activated, the cells undergo late-stage apoptosis, and apoptotic cell death is inevitable [55]. To discriminate between early and late apoptosis of cells, a flow-cytometric based Annexin V/propidium iodide (PI) Cell Apoptosis kit was used to detect the apoptotic rate of cells after SC, SCA, SCH, and SCAH treatments. According to the data shown in Figure 7, late apoptosis of cells predominated in SC-, SCA-, SCH-, and SCAH-induced cell death. There were statistically significant increases from 14.0% ± 0.3% (control) to 40.0% ± 0.1% (SC), 45.9% ± 1.1% (SCA), 39.7% ± 1.2% (SCH), and 27.7% ± 0.8% (SCAH), in the percentages of late apoptotic cells, and significant reductions from 71.4% ± 0.6% (control) to 44.3% ± 0.9% (SC), 37.9% ± 0.5% (SCA), 39.1% ± 0.4% (SCH), and 55.2% ± 2.7% (SCAH), in the percentages of live cells. Among the degraded fucoidans, SCA induced the largest number of late apoptotic cells. In addition, the percentage of live cells in the non-treated control was 71.4% ± 0.6%, suggesting that A-549 cells alone also showed ongoing apoptotic and necrotic effects after 48 h of starvation. Further investigations regarding the optimal treatment conditions satisfying both the control and the experimental group are needed. The in vitro anti-lung cancer potential of fucoidan was examined previously by other investigators. A-549 cells were exposed to fucoidan extracted from *Padina distromatica* at 1000 μg/mL for 48 h and showed a survival rate of approximately 65.44% ± 1.35% [59]. Compared with *P. distromatica* fucoidan, the cytotoxic effect of SCA to A-549 cells was higher (treatment of A-549 cells at 300 μg/mL for 48 h resulted in a survival rate of 36.5% ± 3.9%, Figure 2A). In addition, the fucoidan extracted from *Turbinaria conoides* exhibited a survival rate of approximately 35% when used to treat A-549 cells at 250 μg/mL for 72 h [60]; therefore, in general, it also showed that the cytotoxic effect of SCA to A-549 cells was higher than *T. conoides* fucoidan, since the treating concentration was similar but the treatment duration of SCA was shorter (48 h) (Figure 2A). These data suggest that SCA may serve as a good anti-lung cancer candidate as compared to other reported data. Moreover, Boo et al. utilized commercialized *Undaria pinnatifida* fucoidan (obtained from Sigma) and determined that fucoidan induces apoptosis in A-549 cells via caspase-9 activation, cleavage of poly-ADP-ribose polymerase (PARP), and activation of extracellular signal-regulated kinases (ERK) 1/2, as well as downregulation of phospho-p38 and phospho- phosphatidylinositol 3-kinase (PI3K)/Akt expression [61]. In the present study, we utilized fucoidan from *S. crassifolium* and its degraded products, and we revealed that these fucoidan extracts showed apoptotic effects on A-549 cells via loss of MMP, decreased Bcl-2 expression, increased cytochrome c release, increased active caspase-9 and -3, and augmented late apoptosis of A-549 cells. These data provide evidence that fucoidan and its degraded products induced apoptosis of A-549 cells. Taken together, SC-, SCA-, SCH-, and SCAH-induced cell death was found to involve mitochondrial-dependent apoptosis as elucidated by the loss of MMP, decreased Bcl-2 expression, increased release of cytochrome c, increased activation of caspase-9 and -3, and increased late apoptosis of cells. Generally, degraded fucoidans had a more potent effect in terms of inducing apoptosis of A-549 cells compared with SC. Since SCA has a high amount of low-molecular-weight fraction, the highest fucose content, is highly cytotoxic to A-549 cells, has a low cytotoxic effect on BEAS-2B cells, and exhibits the highest ability to suppress Bcl-2 expression, induce cytochrome c release, activate caspase-9, and promote late apoptosis of A-549 cells; thus, it can be recommended as a potential therapeutic agent for preventive or auxiliary treatment of lung cancer.

### 2.10. The Akt/mTOR Pathway Is Involved in SC-, SCA-, SCH-, and SCAH-Induced Apoptosis of A-549 Cells

**Figure 8:**
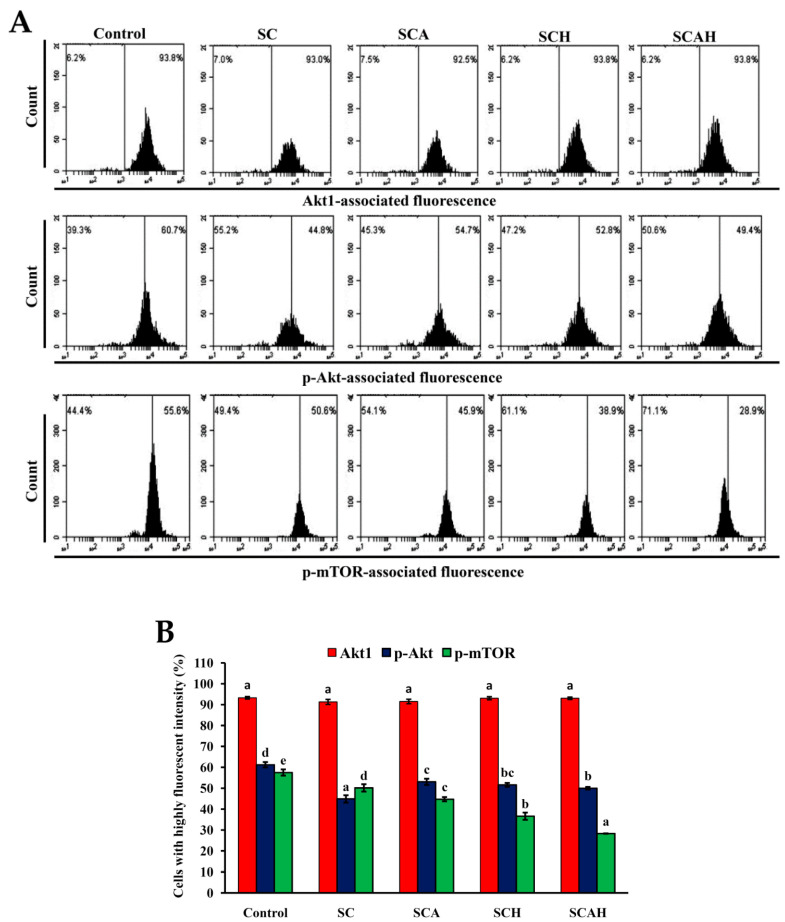
Effects of SC, SCA, SCH, and SCAH treatments on the levels of Akt serine/threonine kinase 1 (Akt1), p-Akt, and p-mammalian target of rapamycin (mTOR) in A-549 cells. Flow-cytometric analysis of A-549 cells treated without and with 200 μg/mL SC, SCA, SCH, and SCAH for 48 h: (**A**) histograms; (**B**) bar chart. Bars in the same Akt1, p-Akt, or p-mTOR group bearing different letters (in a, b, c, d, and e) significantly differ at the level of 0.05. Experiments were repeated three times.

Previous studies indicated that the Akt/mammalian target of rapamycin (mTOR) pathway is closely related to a variety of cellular functions including adhesion, invasion, proliferation, angiogenesis, migration, and survival [62]. In addition, the Akt/mTOR signaling cascade is frequently activated in diverse cancers [63,64,65]. As the Akt/mTOR pathway is frequently activated in cancer, numerous research efforts sought to target this pathway with a view to developing pharmacologic interventions for various cancers, such as colon cancer [66], endometrial cancer [67], breast cancer [68], prostate cancer [69], human small-cell lung cancer cells [70], non-small-cell lung cancer [71], and ovarian cancer [72]. In the present study, a flow-cytometric approach was conducted to evaluate the expression levels of p-Akt, Akt serine/threonine kinase 1 (Akt1), and p-mTOR in A-549 cells treated with SC, SCA, SCH, or SCAH at a dose of 200 μg/mL for 48 h. The results presented in Figure 8 suggest that all of the tested fucoidans suppressed levels of p-Akt and p-mTOR as compared to the untreated control. In addition, it was noted that the expression of total Akt (Akt1) did not vary among the different treatments. Our results are similar to previous findings indicating that the commercialized fucoidan extract from *Fucus vesiculosus* suppressed levels of p-Akt and p-mTOR of A-549 cells dose- and time-dependently [73], and commercialized *U*. *pinnatifida* fucoidan induced apoptosis in A-549 cells via downregulation of phospho-p38 and phospho-PI3K/Akt expression [61]. In summary, the data presented here suggest that Akt/mTOR signaling may play a role in the death of A-549 cells induced by SC, SCA, SCH, and SCAH. Further investigations are needed to elucidate the precise mechanisms and to explore targeting of signaling pathways in lung cancer, especially using in vivo models.

## 3. Materials and Methods

### 3.1. Materials

Samples of *S. crassifolium*, a type of brown seaweed, were collected from Kenting (Pingtung, Taiwan), and, after washing and drying, they were sealed in aluminum foil bags and kept at 4 °C until use. Dextrans (5, 50, 150, and 670 kDa), l-fucose, d-glucuronic acid, dimethyl sulfoxide (DMSO), potassium bromide (KBr), 2,2,2-trifluoroacetic acid (TFA), and 3-(4,5-dimethylthiazol-2-yl)-2,5-diphenyltetrazolium bromide (MTT) were obtained from Sigma-Aldrich (St. Louis, MO, USA). Ham’s F12K medium, Dulbecco’s Modified Eagle Medium (DMEM) medium, trypsin/ ethylenediaminetetraacetic acid (EDTA), fetal bovine serum (FBS), penicillin, and streptomycin were obtained from Gibco Laboratories (Grand Island, NY, USA). TMRE and fluorescein isothiocyanate (FITC)-labeled anti-Bcl-2 antibodies were obtained from Molecular Probes, Invitrogen Corp. (Carlsbad, CA, USA).

### 3.2. Extrusion Process

The extrusion process was done according to the method reported by Huang et al. [15] with minor modification. In brief, a single-screw laboratory extruder (Tsung Hsing Co. Ltd., Kaohsiung, Taiwan) equipped with screw diameter 74 mm, L/D ratio 3.07:1, and rounded die head with a diameter of 5 mm was used. The algal sample was used as the raw material and was preconditioned to a moisture content of 35%. The feed rate was constant at 10.4 kg/h. Other extrusion parameters were set with barrel temperature at 115 °C and screw speed at 360 rpm. The algal extrudate was dried at 55 °C for 30 min, cooled to room temperature (RT), ground into particles that could pass through a 20-mesh screen, and stored at 4 °C for further extraction experiments.

### 3.3. Water Extraction Procedure

The extraction of native fucoidan from *S. crassifolium* was done according to the method reported by Huang et al. [23]. In brief, the algal sample was mixed with 95% ethanol (*w*/*v* = 1:10), shaken for 1 h at room temperature to remove pigments and lipid, and then centrifuged at 970× *g* for 10 min. The residue was then collected, mixed with double distilled water (*w*/*v* = 1:10), and placed in a water bath kept at 85 °C for 1 h with shaking to extract the polysaccharides. The mixture was centrifuged at 3870× *g* for 10 min, and the supernatant was collected. Ethanol (95%) was added into the supernatant to give a final ethanol concentration of 20% in order to precipitate alginic acid. The mixture was centrifuged at 9170× *g* for 30 min, the supernatant was collected, and 95% ethanol was added until a final ethanol concentration of 50% was reached in order to obtain fucoidan precipitate. The ethanol-precipitated fucoidan was then recovered by centrifugation at 9170× *g* for 30 min, dried at 40 °C, milled, and stored at 4 °C for further use. Extraction yield was calculated using the following equation:

Extraction yield (%) = (weight of the extracted solid, dry basis/weight of the sample, dry basis) × 100(1)

### 3.4. Preparation of Degraded Fucoidans

A sample of native fucoidan weighing 0.2 g was dissolved into 20 mL of distilled water and mixed with 10 mmol/L AA, 10 mmol/L H_2_O_2_, or a mixed solution of 10 mmol/L H_2_O_2_ and 10 mmol/L AA. The native fucoidan was degraded at RT for 16 h. Then, the degraded fucoidan was precipitated using 75% ethanol, collected, and dried for further use [24].

### 3.5. Intrinsic Viscosity Analysis

The viscosity measurements were performed using an Ubbelohde viscometer at 25 ± 1 °C. The pure solvent was ddH_2_O, and the intrinsic viscosity (*η*_r_) was determined using the following equation:

*η*_r_ = (ln*t*/*t*_0_)/*c* (2)

where *t* = efflux time for solution (s), *t*_0_ = efflux time for the pure solvent (s), and *c* = concentration of solution (g/mL).

### 3.6. Molecular Weight Analysis

The average molecular weights of the fucoidans were determined using a size-exclusion HPLC column Superdex 200 (300 mm × 10 mm inner diameter (ID), GE Healthcare, Piscataway, NJ, USA) using a Shimadzu HPLC system (Shimadzu, Kyoto, Japan) equipped with a refractive index detector. The chromatography conditions were as follows: eluent 0.2 M NaCl; flow rate 0.4 mL/min, sample concentration 10 mg/mL; injection volume 0.15 mL; temperature 25 °C. The column was calibrated with dextrans of different molecular weight (5, 50, 150, and 670 kDa).

### 3.7. Analytical Methods

The fucose was estimated using the protocol described by Gibbons [74], and l-fucose was utilized as the standard. Total sugar content was assayed using a phenol–sulfuric acid method using l-fucose as the standard. The sulfate content was determined according to the protocol described by Yang et al. [22]. Polyphenols were analyzed by the Folin–Ciocalteu method, and gallic acid was used as the standard [75].

### 3.8. Monosaccharide Composition Analysis

The monosaccharide composition of fucoidan was determined according to a previously reported method [23] using six monosaccharides (l-fucose, d-galactose, d-glucuronic acid, d-galacturonic acid, d-xylose, and d-mannose) as standards.

### 3.9. FTIR Spectroscopy

The FTIR spectra were analyzed according to a protocol described elsewhere [41] using an FT-730 instrument (Horiba, Kyoto, Japan). A KBr pellet was prepared by mixing 1 mg of fucoidan with 50 mg of potassium bromide. The spectrum was read between 400 and 4000 cm^−1^.

### 3.10. NMR Spectroscopy

The polysaccharide sample was dissolved with 99.9% D_2_O in an NMR tube, and the NMR spectra were read on a Varian VNMRS-700 NMR spectrometer (Varian, Lexington, MA, USA).

### 3.11. Cell Culture

BEAS-2B (human bronchial epithelial cells, ATCC CRL-9609, Manassas, VA, USA) and A-549 (human lung carcinoma, BCRC 60074, Hsinchu, Taiwan) were obtained from the ATCC (American Type Culture Collection) (Manassas, VA, USA) and the BCRC (Bioresource Collection and Research Center) (Hsinchu, Taiwan), respectively. A-549 cells were cultured in complete Ham’s F12K medium and BEAS-2B was maintained in complete DMEM medium. The complete medium was prepared by supplementing plain medium with 10% FBS, 100 µg/mL streptomycin, and 100 units/mL penicillin. All cells were cultured in a 37 °C humidified 5% CO_2_ atmosphere. The cells were passaged every 2–3 days.

### 3.12. Evaluation of Cytotoxic Activity

The cytotoxic activity of the fucoidan extracts was measured using the MTT assay. Briefly, cells were cultured in growth medium with 37 °C humidified 5% CO_2_ atmosphere for 24 h. All fucoidan extracts were prepared as a 20 mg/mL stock solution by thoroughly dissolving fucoidan powder in phosphate-buffered saline (PBS). The medium was then removed, and the cells were treated with various concentrations of fucoidan extracts. Final concentrations of 0, 50, 100, 200, 300, 400, and 500 μg/mL were obtained by diluting the stock solution with serum-free medium to prevent the fucoidan extract from potentially losing its potency in the presence of serum. After 48 h of treatment, cells were washed with PBS once, and MTT stock solution was added to each culture so that the final concentration of MTT in the medium was 0.1 mg/mL. After 2–4 h of incubation, the formazan was solubilized by adding isopropanol and measured by absorption at 560 nm. The cell viability was expressed as a percentage of MTT reduction.

### 3.13. Flow Cytometry-Based Analyses

In all flow cytometry-based analyses, cells (4 × 10^4^ cells/mL) were incubated without (as a non-treated control, cells were in serum-free medium) and with 200 μg/mL SC, SCA, SCH, or SCAH (these fucoidan extracts were prepared as a 20 mg/mL stock solution by thoroughly dissolving fucoidan powder in PBS; a final concentration of 200 μg/mL was obtained by diluting the stock solution with serum-free medium) for 48 h, and then cells were de-attached by trypsin and rinsed two times in cold PBS to obtain cell samples. Then, each flow cytometry-based analysis was performed according to the protocols below.

For the MMP analysis, the assay was conducted according to the method of Yang et al. [22]. Briefly, single-cell suspensions were washed twice with PBS and incubated, in the dark, for 20 min at 37 °C with TMRE (100 nM). After labeling, cells were washed and re-suspended for flow-cytometric measurement in staining solution.

For Bcl-2 expression analysis, single-cell suspensions were fixed using fixation buffer at 37 °C for 20 min. Then, the cells were permeabilized using permeabilization buffer and incubated, in the dark, for 1 h at RT with FITC-labeled anti-Bcl-2 antibody (1:25, *v*/*v*). After labeling, cells were washed and re-suspended for flow-cytometric measurement in staining solution.

The analysis of cytochrome c release was conducted by following the method of Huang et al. [41]. Briefly, single-cell suspensions were fixed using fixation buffer at 37 °C for 20 min. Then, the cells were permeabilized using permeabilization buffer and incubated, in the dark, for 1 h at RT with FITC-labeled anti-cytochrome c antibody (1:10, *v*/*v*). After labeling, cells were washed and re-suspended for flow-cytometric measurement in staining solution.

For activated caspase -9 and -3 analyses, the method of Huang et al. was used [41]. Briefly, single-cell suspensions were incubated, in the dark, for 1 h at 37 °C with FITC/LEHD/FMK solution (for caspase-9 detection) or FITC/DEVD/FMK solution (for caspase-3 detection). After labeling, cells were washed and re-suspended for flow-cytometric measurement in staining solution.

The annexin V-FITC/PI staining analysis was conducted using an annexin V-FITC apoptosis detection kit according to the method of Yang et al. [22]. Briefly, single-cell suspensions were incubated, in the dark, for 15 min at RT with annexin V-FITC (1:20, *v*/*v*) and PI (1:20, *v*/*v*). After labeling, cells were washed and re-suspended for flow-cytometric measurement in staining solution.

For phosphorylated Akt and mTOR analyses, the assays of phosphorylated Akt and mTOR were conducted using the method of Huang et al. [76]. In brief, single0cell suspensions were fixed using fixation buffer at 37 °C for 1 h. Then, the cells were incubated, in the dark, for 1 h at RT with allophycocyanin (APC)-conjugated anti-Akt1 antibody (1:50, *v*/*v*), FITC-conjugated anti-phospho-Akt (Ser473) antibody (1:20, *v*/*v*), or phycoerythrin (PE)-conjugated anti-phospho-mTOR (Ser2448) antibody (1:20, *v*/*v*). After labeling, cells were washed and re-suspended for flow-cytometric measurement in staining solution. All of the flow-cytometric analyses described above were conducted using a BD Accuri C6 flow cytometer (San Jose, CA, USA).

### 3.14. Statistical Analysis

All data are expressed as means ± SD (*n* = 3). Comparisons between different groups were performed by ANOVA followed by the Duncan multiple range test or the Student’s *t*-test. A *p*-value <0.05 was considered statistically significant.

## 4. Conclusions

In this paper, a native fucoidan (SC) was extracted from *S. crassifolium* pretreated by single-screw extrusion. The extrusion pretreatment process augmented the extraction yield of fucoidan as compared to the non-extruded sample. Three degraded fucoidans (SCA, SCH, and SCAH) were obtained by degrading SC with different combinations of degradation reagents. Among SC, SCA, SCH, and SCAH, the chemical compositions varied but their structural features were similar, as illustrated by the results of FTIR and NMR analyses. In vitro anti-lung cancer studies revealed that SC, SCA, SCH, and SCAH decreased MMP, decreased Bcl-2 expression, increased the release of cytochrome c, increased activation of caspase-9 and -3, and increased late apoptosis of A-549 cells. In general, SCA has a high amount of low-molecular-weight fraction and the highest fucose content, is highly cytotoxic to A-549 cells, and shows a high ability to suppress Bcl-2 expression, to induce cytochrome c release, to promote activation of caspase-9, and to induce late apoptosis of A-549 cells. Therefore, SCA demonstrated excellent potential for development as an adjuvant treatment of lung cancer. Additional in vitro experiments showed that the Akt/mTOR signaling pathway is involved in SC-, SCA-, SCH-, and SCAH-induced apoptosis of A-549 cells. Further experiments are warranted to elucidate the precise signaling mechanisms involved, especially using in vivo studies.

## Figures and Tables

**Table 1:** Viscosity, molecular weight, and composition analyses of *Sargassum* *crassifolium* fucoidan (SC) and that degraded by ascorbic acid (SCA), hydrogen peroxide (SCH), and their combination (SCAH).

Viscosity	SC ^3^	SCA ^3^	SCH ^3^	SCAH ^3^
Intrinsic viscosity (mL/g)	113.9 ± 2.4 ^d^	78.6 ± 4.2 ^b^	102.5 ± 0.9 ^c^	36.9 ± 2.6 ^a^
**Molecular Weight (MW)**	**SC**	**SCA**	**SCH**	**SCAH**
Peak 1 (Peak MW ^1^ (kDa))	427.8	455.1	427.8	487.1
Peak 1 (MW interval (kDa))	188.7–1064	216.2–898.9	194.3–998.1	264.5–956.4
Peak 1 (Peak area (%))	100.0	50.3	61.0	49.2
Peak 2 (Peak MW (kDa))	ND ^4^	3.06	3.14	3.11
Peak 2 (MW interval (kDa))	ND	1.65–9.95	1.90–15.54	1.66–12.55
Peak 2 (Peak area (%))	ND	49.7	39.0	50.8
**Chemical Composition**	**SC**	**SCA**	**SCH**	**SCAH**
Total sugar (%) ^2^	45.58 ± 0.80 ^d^	41.70 ± 0.91 ^c^	30.83 ± 0.21 ^a^	33.83 ± 0.71 ^b^
Fucose (%) ^2^	27.31 ± 1.59 ^b^	35.22 ± 2.79 ^c^	20.08 ± 1.68 ^a^	30.08 ± 3.11 ^b^
Sulfate (%) ^2^	18.64 ± 1.43 ^b^	13.67 ± 2.19 ^a^	19.23 ± 0.83 ^b^	20.39 ± 3.28 ^b^
Polyphenols (%) ^2^	1.85 ± 0.07 ^c^	1.29 ± 0.02 ^b^	1.17 ± 0.02 ^a^	1.12 ± 0.01 ^a^
**Monosaccharide Composition (Molar Ratio)**	**SC**	**SCA**	**SCH**	**SCAH**
Fucose	1	1	1	1
Galactose	0.24	0.30	0.27	0.28
Glucuronic acid	0.19	0.01	0.07	0.03
Galacturonic acid	0.15	0.11	0.06	0.05
Mannose	0.08	0.05	0.07	0.06
Xylose	0.04	0.05	0.10	0.02

^1^ Peak MW: molecular weight of the highest peak. ^2^ Total sugars (%), fucose (%), sulfate (%), and polyphenols (%) = (*g*/*g*, dry basis) × 100. ^3^ Experiments were performed in triplicate; values in the same row with varying letters (in ^a^, ^b^, ^c^, and ^d^) differ (*p* < 0.05). ^4^ ND: not detected.

## Supplementary Materials

**^1^**H-NMR spectra and ^13^C-NMR spectra for SC, SCA, SCH, and SCAH are available online at https://www.mdpi.com/1660-3397/18/6/334/s1.
