# Supplementary material for: Degradation of Sargassum crassifolium Fucoidan by Ascorbic Acid and Hydrogen Peroxide, and Compositional, Structural, and In Vitro Anti-Lung Cancer Analyses of the Degradation Products
Source: Mar Drugs. 2020 Jun 26;18(6):334. doi: 10.3390/md18060334 (PMC7345171; doi:10.3390/md18060334)
Supplement: Supplementary file 1 [file marinedrugs-18-00334-s001.pdf]

Supplementary Information

Figure S1. NMR analyses of SC, SCA, SCH, and SCAH. (**A**) ^{1}H-NMR spectra for SC, SCA, SCH, and SCAH. (**B**) ^{13}C-NMR spectra for SC, SCA, SCH, and SCAH. The characteristic peaks are indicated in each graph.

Figure S1. NMR analyses of SC, SCA, SCH, and SCAH. (**A**) ^{1}H-NMR spectra for SC, SCA, SCH, and SCAH. (**B**) ^{13}C-NMR spectra for SC, SCA, SCH, and SCAH. The characteristic peaks are indicated in each graph.

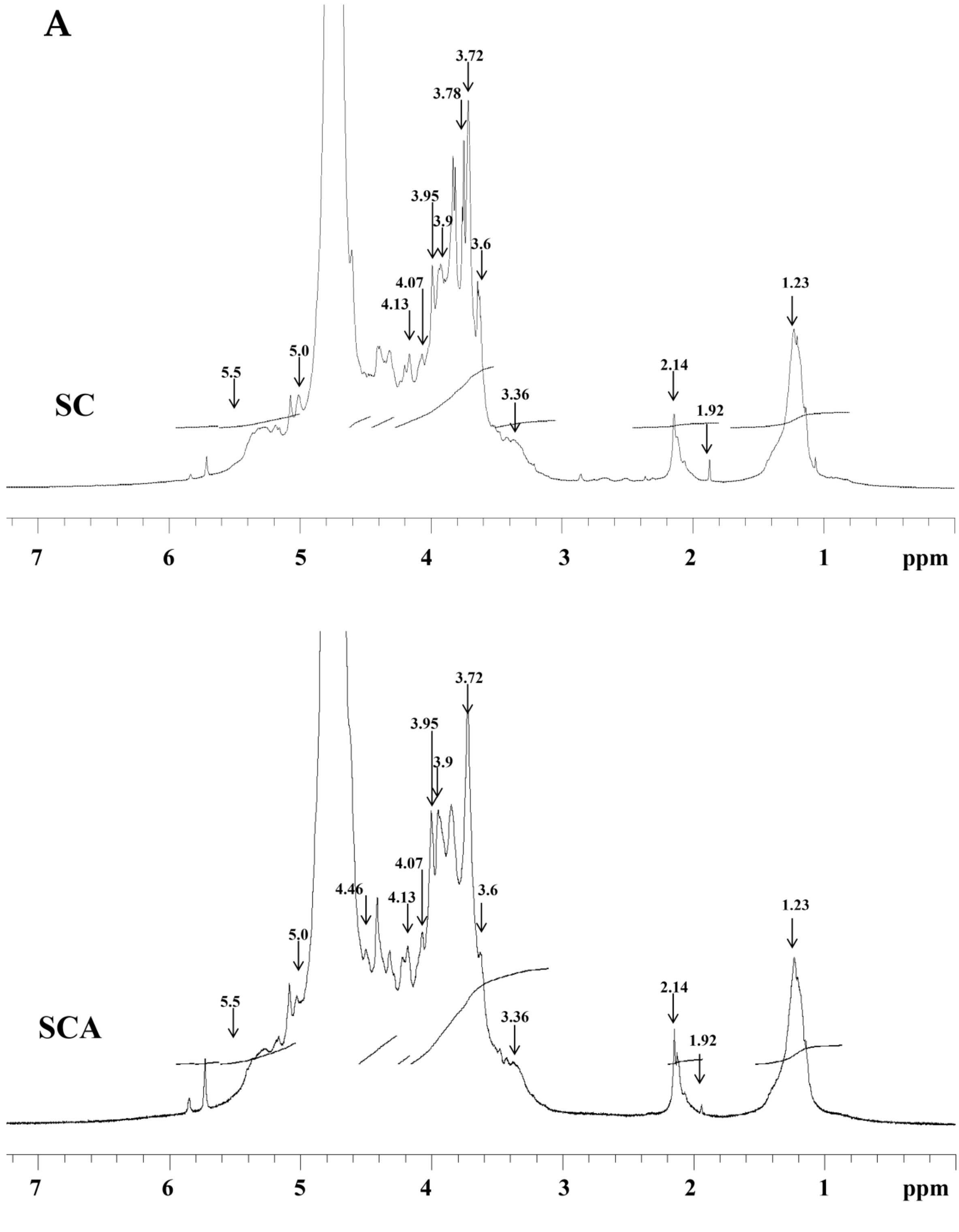

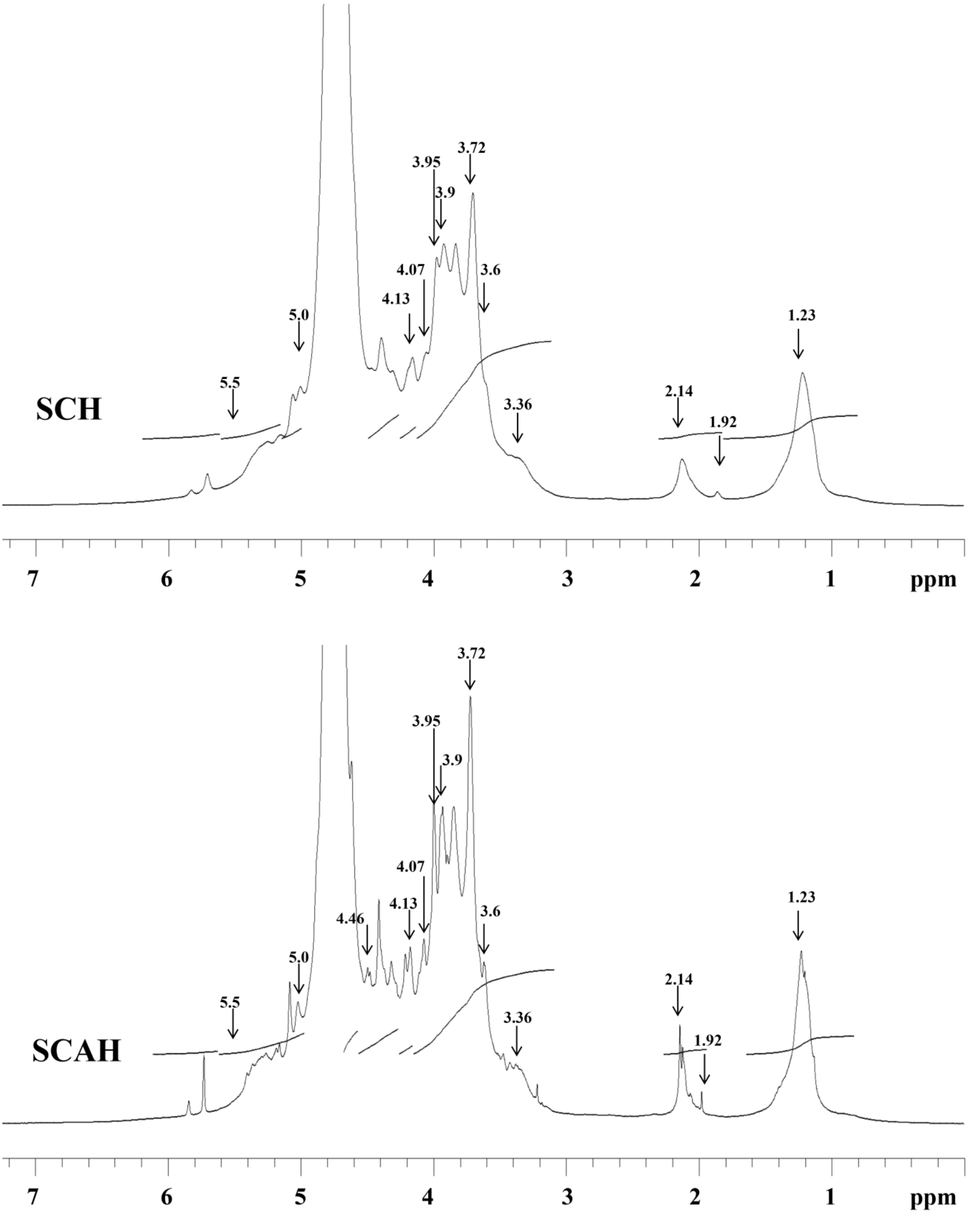
SCH
5.5
5.0
4.13
4.07
3.95
3.9
3.72
3.6
3.36
2.14
1.92
1.23
7 6 5 4 3 2 1 ppm
SCAH
5.5
5.0
4.46
4.13
4.07
3.95
3.9
3.72
3.6
3.36
2.14
1.92
1.23
7 6 5 4 3 2 1 ppm

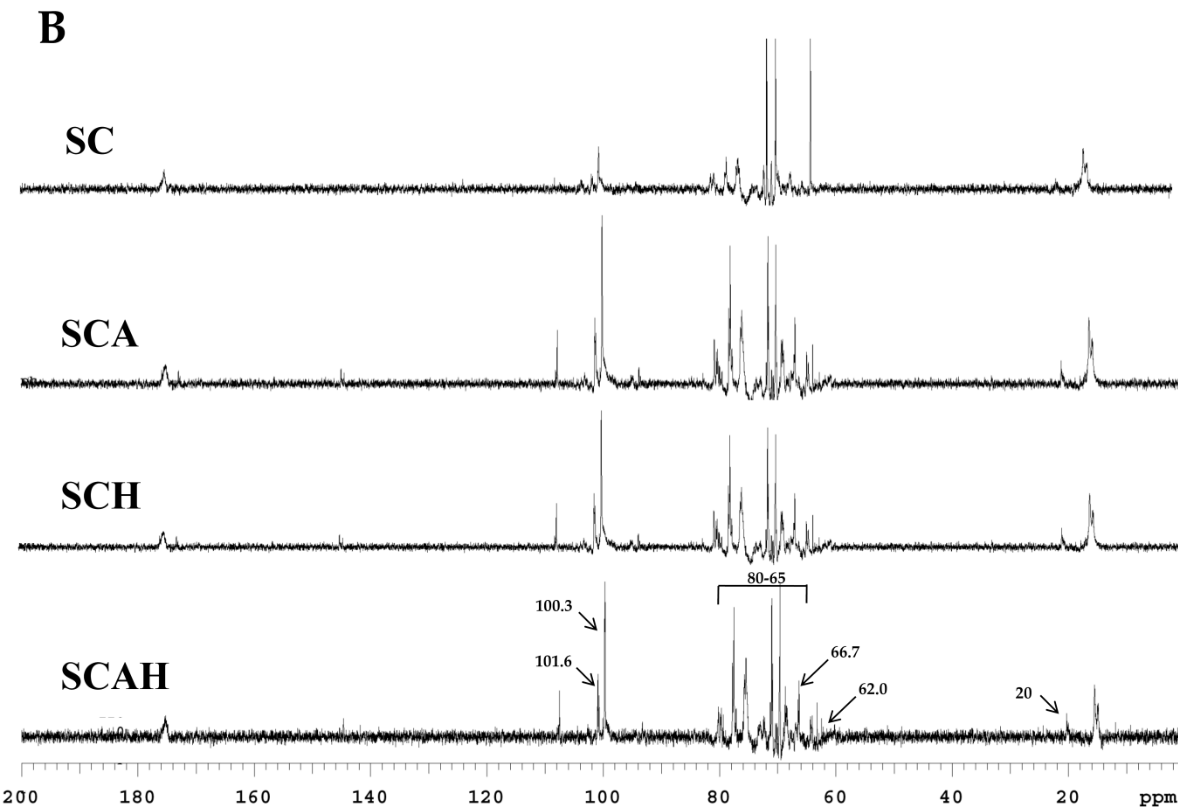

B
SC
SCA
SCH
SCAH
100.3
101.6
80-65
66.7
62.0
20
200
180
160
140
120
100
80
60
40
20
ppm
